# Identification of CD36 as a new interaction partner of membrane NEU1: potential implication in the pro-atherogenic effects of the elastin receptor complex

**DOI:** 10.1007/s00018-018-2978-6

**Published:** 2018-11-29

**Authors:** Charlotte Kawecki, Olivier Bocquet, Christian E. H. Schmelzer, Andrea Heinz, Christian Ihling, Amandine Wahart, Béatrice Romier, Amar Bennasroune, Sébastien Blaise, Christine Terryn, Kenneth J. Linton, Laurent Martiny, Laurent Duca, Pascal Maurice

**Affiliations:** 1 UMR CNRS 7369 Matrice Extracellulaire et Dynamique Cellulaire (MEDyC), Team 2 “Matrix Aging and Vascular Remodelling”, Université de Reims Champagne Ardenne (URCA), UFR Sciences Exactes et Naturelles, Moulin de la Housse, BP1039, 51687 Reims Cedex 2, France; 2 Fraunhofer Institute for Microstructure of Materials and Systems IMWS, Halle (Saale), Germany; 3 Institute of Pharmacy, Faculty of Natural Sciences I, Martin Luther University Halle-Wittenberg, Halle (Saale), Germany; 4 Department of Pharmacy, University of Copenhagen, Copenhagen, Denmark; 5 PICT Platform, Université de Reims Champagne Ardenne (URCA), Reims, France; 6 Blizard Institute, Barts and the London School of Medicine, Queen Mary University of London, London, UK

**Keywords:** NEU1, Elastin-derived peptides, Sialylation, CD36, Atherosclerosis

## Abstract

In addition to its critical role in lysosomes for catabolism of sialoglycoconjugates, NEU1 is expressed at the plasma membrane and regulates a myriad of receptors by desialylation, playing a key role in many pathophysiological processes. Here, we developed a proteomic approach dedicated to the purification and identification by LC–MS/MS of plasma membrane NEU1 interaction partners in human macrophages. Already known interaction partners were identified as well as several new candidates such as the class B scavenger receptor CD36. Interaction between NEU1 and CD36 was confirmed by complementary approaches. We showed that elastin-derived peptides (EDP) desialylate CD36 and that this effect was blocked by the V14 peptide, which blocks the interaction between bioactive EDP and the elastin receptor complex (ERC). Importantly, EDP also increased the uptake of oxidized LDL by macrophages that is blocked by both the V14 peptide and the sialidase inhibitor 2-deoxy-2,3-didehydro-*N*-acetylneuraminic acid (DANA). These results demonstrate, for the first time, that binding of EDP to the ERC indirectly modulates CD36 sialylation level and regulates oxidized LDL uptake through this sialidase. These effects could contribute to the previously reported proatherogenic role of EDP and add a new dimension in the regulation of biological processes through NEU1.

## Introduction

Sialidases, or neuraminidases, represent a family of exoglycosidases that remove terminal sialic acid residues from glycoproteins, glycolipids and oligosaccharides. The human sialidase family comprises four members: NEU1, NEU2, NEU3 and NEU4 [1]. Although initially described as a lysosomal sialidase, it is now clear from many reports that NEU1 is also expressed at the plasma membrane of cells and regulates a plethora of membrane receptors by desialylation [2] such as the toll-like receptor 4 (TLR4) [3], the TrkA tyrosine kinase receptor [4] and the insulin receptor [5]. As exemplified for TLR4, desialylation of the receptor by NEU1 is essential for receptor activation, clustering, and cellular signaling in dendritic and macrophage cells [3]. It has further been demonstrated that activation of ligand-induced TLR4 and subsequent signaling pathways may be brought by binding of complexes of NEU1 and matrix metalloprotease-9 (MMP-9) to TLR4 at the plasma membrane [6]. At the plasma membrane, NEU1 also associated with the elastin-binding protein (EBP) and the carboxypeptidase protective protein/cathepsin A (PPCA) forming the elastin receptor complex (ERC) which is required for elastogenesis and signal transduction through this receptor [7–9]. Consequently, NEU1 emerged not only as a catabolic enzyme but also as a key regulator of cell signaling and receptor activation. By its ability to interact with membrane receptors and to modulate their sialylation levels, NEU1 is a critical regulator of several biological processes such as cell proliferation, differentiation, elastogenesis and inflammation [2]. It has been further proposed that NEU1 may have important roles in vivo in tumorigenesis [10, 11] and in the biological effects mediated by the elastin-derived peptides (EDP) in age-related vascular diseases [5, 12, 13]. Inhibition of NEU1 by the sialidase inhibitor 2-deoxy-2,3-didehydro-N-acetylneuraminic acid (DANA) decreases production of reactive oxygen species (ROS) and reduces migration of mouse monocytes in response to EDP. Chimeric LDLR^−/−^ mice devoid of NEU1 activity in their hematopoietic lineage also show reduced atherosclerosis [13]. Together, these data demonstrate that NEU1 may be a critical regulator of monocyte/macrophage functions. This has been further strengthened by the recent demonstration of a positive feedback loop between IL-1β, LPS and NEU1 in monocytes and macrophages that may promote atherosclerosis by enhancing a pro-inflammatory state [14].

In the present study, we investigated new roles for plasma membrane NEU1 sialidase in monocyte/macrophage functions, by developing a proteomic approach to purify and identify by mass spectrometry the interaction partners of NEU1 in macrophages differentiated from THP-1 cells. We identified known interaction partners of NEU1 and several new potential candidates such as the class B scavenger receptor CD36. Interaction between NEU1 and CD36 at the plasma membrane was confirmed by complementary approaches such as co-immunoprecipitation and Förster resonance energy transfer (FRET), and led to functional consequences for CD36 sialylation and the uptake of oxidized LDL (oxLDL) by macrophages.

## Materials and methods

### Materials

The plasmid-encoding human PPCA was kindly provided by Pr Alessandra d’Azzo and is described elsewhere [15]. The plasmid-encoding NEU1-Flag and NEU1-HA constructs were obtained as detailed previously [16]. The plasmid-encoding human CD36 (pCI-CD36–12His) has been described previously [17]. The cDNA-encoding monomeric teal protein in a pcDNA3 vector was kindly provided by Pr Maddy Parsons [18]. Site-directed mutagenesis (Quikchange, Agilent) was used to engineer a *Nhe*I restriction enzyme site upstream of the teal cDNA and a *Bst*EII site in the coding sequence for a poly-glycine protein linker region that precedes the teal cDNA. The cDNA for CD36 was excised from pCI-CD36-12His using *Nhe*I and *Bst*EII and subcloned into the equivalent sites in pcDNA3-Teal to generate pCD36-Teal. The construct includes a consensus human ribosome binding site at the CD36 start codon and creates an in-frame fusion of CD36 with the poly-glycine linker and teal, such that the primary sequence of the product now reads: CD36-SKTIKghlggggsggggssgVSKGEETT-Teal. The NEU1-YFP construct was obtained using human NEU1 cDNA as template and the Phusion High-Fidelity DNA Polymerase (ThermoScientific). Restriction sites for *Hind*III and *BamH*I were introduced by PCR. After digestion by the respective restriction enzymes, the resulting inset (NEU1) was ligated into a pcDNA3 vector encoding the YFP protein at the C terminus of the fusion protein. All cDNA sequences were confirmed by sequencing. EZ-Link^®^ sulfo-NHS-LC-biotin and monomeric avidin agarose beads were purchased from ThermoScientific. Free biotin, mouse monoclonal anti-Flag M2 antibodies and 2-O-(*p*-nitrophenyl)-α-d-*N*-acetylneuraminic acid were from Sigma. Protein G Sepharose beads and streptavidin beads were purchased from GE healthcare. 2′-(4-Methylumbelliferyl)-alpha-d-*N*-acetylneuraminic acid was from BioSynth and 2-deoxy-2,3-didehydro-*N*-acetylneuraminic acid (DANA) and phorbol-12-myristate-13-acetate (PMA) from Calbiochem. Biotinylated *Sambucus Nigra* Agglutinin (SNA) was purchased from Vector laboratories and Dil-oxLDL from Alfa Aesar. The DIG glycan differentiation kit was from Roche. The V14 peptide (VVGSPSAQDEASPL) was synthesized by Genecust with 99% purity. Rabbit polyclonal anti-NEU1 (H-300) antibodies were purchased from Santa Cruz and mouse monoclonal anti-CD36 antibodies from Santa Cruz and STEMCELL Technologies. Rabbit polyclonal anti-NEU3 were from Invitrogen. Rabbit monoclonal anti-HA and mouse monoclonal anti-β_2_-integrin antibodies were from Cell Signaling and Chemicon, respectively. Alexa Fluor 488 or 568-conjugated donkey anti-mouse or rabbit antibodies were from Invitrogen.

### Kappa-elastin preparation

EDP were prepared as described previously [7]. Briefly, insoluble elastin was prepared from bovine *ligamentum nuchae* by hot alkali treatment. Purity was assessed by comparing its amino acid composition to the one predicted from the elastin gene product. Soluble EDP were then obtained from insoluble elastin as described [7]. The obtained mixture of EDP, termed kappa-elastin (kE), has been shown to contain several peptides harboring the bioactive motif GxxPG [5]. The composition of EDP from kE was compared to EDP obtained after proteolysis of human elastin by neutrophil elastase by mass spectrometry analysis and shown to contain similar peptides harboring the bioactive GxxPG motif [5]. In addition, kE has been shown to exhibit the same biological properties as elastin hydrolysates obtained by human neutrophil elastase as both EDP mixtures increase pro-MMP1 production in human skin fibroblasts with comparable extent [5, 19].

### Cell culture and transient overexpression of NEU1

COS-7 cells were cultured in Dulbecco’s modified Eagle’s medium supplemented with 10% heat-inactivated fetal bovine serum, 100 units/mL penicillin, 0.1 mg/mL streptomycin at 37 °C in a humidified atmosphere at 95% air and 5% CO_2_. This cell line has been widely used to overexpress NEU1 [7, 16, 20–25]. Transient transfections were performed with JET-PEI (Polyplus Transfection), according to the manufacturer’s protocol, and experiments were performed 48 h post-transfection. The human monocytic THP-1 cell line was cultured in RPMI 1640 medium supplemented with 10% heat-inactivated fetal bovine serum, 100 units/mL penicillin and 0.1 mg/mL streptomycin. THP-1 monocytes were differentiated into adherent macrophages using 50 nM PMA (Calbiochem) for 72 h.

### Purification of plasma membrane NEU1 and its interaction partners

Approximatively 4 × 10^7^ COS-7 cells transfected with NEU1-Flag/PPCA (1:2) or 4.8 × 10^8^ macrophages were washed three times in PBS, incubated with 0.5 mg/mL of EZ-Link^®^ sulfo-NHS-LC-biotin for 30 min at 4 °C, and quenched with 100 mM glycine (30 min, 4 °C). Then, the cells were scraped in HEPES buffer (50 mM HEPES, 150 mM NaCl, 2 mM EDTA, protease inhibitor cocktail, 10 mM NaF, 2 mM Na_3_VO_4_, pH 7.5) containing 1% Triton X-100, sonicated and incubated under gentle rotative mixing (3 h, 4 °C) for solubilization of membrane proteins. Lysates were then centrifuged (20,000*g*, 45 min, 4 °C) to pellet insoluble material and supernatants were incubated with monomeric avidin agarose beads for 45 min at 4 °C to purify biotinylated membrane proteins. After several washes, biotinylated proteins were eluted from beads by 10 mM free biotin in HEPES buffer under gentle rotative mixing at 4 °C and NEU1 complexes were immunoprecipitated using rabbit polyclonal anti-NEU1 antibodies pre-adsorbed onto protein G Sepharose beads (overnight, 4 °C). Non-specific interactions were controlled for using rabbit IgG-protein G Sepharose. Immunoprecipitated proteins were eluted with Laemmli buffer and the recovered proteins were denatured in SDS-PAGE loading buffer (62.5 mm Tris/HCl, pH 6.8, 2% SDS, 10% glycerol, 0.5% bromophenol blue) for 10 min at 95 °C. Samples were subjected to SDS-PAGE on a 10% polyacrylamide gel, and proteins were stained by Coomassie blue. For detection of NEU1-Flag, proteins were transferred onto a nitrocellulose membrane. After blocking, membranes were incubated with mouse monoclonal anti-Flag M2 (1/1000, overnight, 4 °C) and immunoreactive bands were revealed using a HRP-conjugated anti-mouse IgG (1/10,000) followed by enhanced chemiluminescence detection reagents (GE Healthcare) and visualized with the Odyssey Fc scanner (LI-COR).

### Protein identification by LC–MS/MS

Each gel lane was systematically excised into 23 bands. Gel bands were diced into small pieces, destained using ammonium bicarbonate and further reduced and alkylated with dithiothreitol and iodoacetamide, respectively. The in-gel digestion was carried out by adding an excess of trypsin solution to each sample and incubation for 14 h at 37 °C. The released peptides were extracted with 50% acetonitrile/0.1% formic acid. The acetonitrile was removed in a Speed Vac and the samples were reconstituted in 0.1% TFA. The peptide mixtures were analyzed on an Ultimate 3000 RSLCnano system coupled to an Orbitrap Fusion Tribrid mass spectrometer (Thermo Fisher Scientific). Briefly, the samples were loaded on the trap column (Acclaim PepMap RP-C18, 300 µm × 5 mm, 5 µm, 100 Å) and washed with water containing 0.1% TFA for 15 min (30 µl min^−1^), before the peptides were separated on the separation column (Acclaim PepMap RP-C18, 75 μm × 250 mm, 2 µm, 100 Å) using gradients from 1 to 35% B (90 min), 35 to 85% B (5 min) followed by 85% B (5 min), with solvent A: 0.1% FA in water and solvent B: 0.08% FA in acetonitrile. MS and MS/MS experiments were performed on the Orbitrap Fusion, which was equipped with a Nanospray Flex Ion Source. Data were acquired in the data-dependent MS/MS mode: Each high-resolution full-scan (automatic gain control (AGC) target value 4 × 10^5^, maximum injection time 50 ms) in the Orbitrap (*m/z* 300–1500, *R* = 120,000) was followed by high-resolution product ion scans in the Orbitrap (collision-induced dissociation, CID), 35% normalized collision energy, *R* = 15,000, AGC target value 5 × 10^4^, maximum injection time 200 ms) within 5 s, starting with the most intense signal in the full-scan mass spectrum (quadrupole isolation window 2 Th). Dynamic exclusion for 60 s (mass window ± 2 ppm) was enabled to allow analysis of less abundant species. Data acquisition was controlled with Xcalibur 3.0.63. Peptides were identified by automated de novo sequencing followed by matching to the UniProt/Swiss-Prot database using the software PEAKS Studio (version 7.5; Bioinformatics Solutions, Waterloo, Canada). The enzyme was set to ‘trypsin’ and a maximum of one missed cleavage was allowed. Carbamidomethylation was considered as fixed modification and oxidation of methionine and deamidation of glutamine and asparagine residues were chosen as variable modifications. Mass error tolerances for precursor and fragment ions were set to 3 ppm and 0.015 Da, respectively. The peptide score threshold was decreased until a false discovery rate of ≤ 2% on the peptide spectrum match level was reached and a minimum of two unique peptides were required for protein identification. Two independent purifications were performed, and protein identifications were compared to a control purification using rabbit IgG. Proteins identified in both conditions were considered as non-specific and discarded.

### Sialidase activity

For the determination of sialidase activity bound to avidin beads, beads were washed with PBS and directly resuspended in a reaction buffer containing 20 mM MES (pH 4.5) and 200 µM 2-O-(*p*-nitrophenyl)-α-d-*N*-acetylneuraminic acid for 4 h at 37 °C in the dark. Reactions were stopped by adding 1 M Na_2_CO_3_ and the absorbance measured using an Infinite F200 PRO microplate reader (TECAN).

Sialidase activity at the plasma membrane of macrophages transfected, or not, with negative control siRNA or NEU1 siRNA, was performed as described previously for human fibroblasts [7]. Cells, seeded in 12-well culture dishes (5 × 10^5^ cells/well), were washed with PBS and incubated with a reaction buffer containing 20 mm CH_3_COONa (pH 6.5) and 400 µM 2′-(4-methylumbelliferyl)-α-d-*N*-acetylneuraminic acid, with or without kE (50 µg/mL), for 2 h at 37 °C in the dark. After incubation, the reaction was stopped by adding 0.4 M glycine buffer (pH 10.4), and the fluorescent 4-methylumbelliferone product released in the medium was measured using the Infinite F200 PRO microplate reader (TECAN).

### NEU1 siRNA gene silencing

Silencer™ predesigned siRNA (ID:8481, Ambion) targeting exon 2 of the human *NEU1* gene were used to silent NEU1 [7, 26]. THP-1 cells (1.5 million) were transfected in 6-well culture dishes with either 50 nM NEU1 siRNA or negative control siRNA (Ambion) using INTERFERin reagent (Polyplus Transfection) according to the protocol provided by the manufacturer. Twenty-four hours after transfection, THP-1 cells were counted, transferred in 12-well culture dishes (500,000/well) and differentiated into adherent macrophages using 50 nM PMA for 72 h before measurement of sialidase activity. GAPDH siRNA (Ambion) was used as a positive control for optimization of transfection conditions and siRNA efficacy was verified at the protein level by Western blotting.

### Immunofluorescence

Macrophages or COS-7 cells transfected with NEU1/PPCA (1:2) and CD36 were grown on sterile coverslips in 24-well plates and stimulated, or not, with kE (50 µg/mL, 1 h). After three washes in PBS, cells were fixed with 2% paraformaldehyde in PBS for 15 min and permeabilized by 0.2% Triton X-100 in PBS for 10 min. After blocking with 10% goat serum in PBS for 1 h, cells were incubated with rabbit polyclonal anti-NEU1 (2 µg/mL) and mouse monoclonal anti-CD36 (2 µg/mL) in PBS containing 1% BSA overnight at 4 °C. Coverslips were then washed three times with PBS and incubated with Alexa Fluor 488-conjugated goat anti-mouse and Alexa Fluor 568-conjugated goat anti-rabbit antibodies (1/1000) in PBS containing 1% BSA for 1 h at room temperature. Coverslips were mounted, visualized with a laser scanning microscope (LSM 710 NLO, Zeiss) and analyzed by Image J software.

### Spectral FRET measurement

COS-7 cells transfected with NEU1-YFP/PPCA (1:2) and/or CD36-Teal were grown on sterile coverslips in 24-well plates and stimulated, or not, with kE (50 µg/mL, 1 h). After three washes in PBS, cells were fixed with 2% paraformaldehyde in PBS for 15 min and washed with PBS. After coverslips mounting, FRET images were acquired using the Laser Scanning Microscope LSM 710 NLO (Zeiss). Samples were excited with 458 nm wavelength (2% power) and emission signal was collected between 420 and 721 nm through 63× oil objective (ON: 1.4) on 32 spectral channels. Image parameters were 512 × 512 pixels, 16 bits and acquisition were done with sum on 16 scans to optimize the signal to noise ratio. Separate images were, respectively, acquired from energy donor alone (CD36-Teal) and energy acceptor alone (NEU1-YFP) samples at 458 nm and 514 nm, respectively, to have the corresponding emission spectra.

### FRET analysis

FRET analysis was based on the protocol described by Leavesley et al. [27]. The first part of spectral images processing was done using ZEN software (Zeiss). Images were filtered with median 3 × 3 and the linear unmixing algorithm with measured references spectra of Teal and YFP were processed. Each pixel of the spectral image can then be described by the following equation: *I*_sp_ = *C*_D_ × *I*_SpD_ + *C*_A_ × *I*_SPA_ + *R*; where *I*_SPD_ and *I*_SPA_ are the donor and acceptor spectra, respectively, *C*_D_ and *C*_A_ are the donor and acceptor spectra contribution, respectively, and *R* is the residue signal which was unidentified by the algorithm. The second part of analysis was done using homemade ImageJ macro to determine FRET efficiency using the following equation: $E_{\text{FRET}} = \frac{{C_{\text{A}} }}{{C_{\text{A}} + C_{\text{D}} }} \times 100$. For each cell, thirty regions of interest (ROI of 0.62 µm^2^) contouring the cell were chosen, and the mean $E_{\text{FRET}}$ was measured.

### Co-immunoprecipitation

COS-7 cells transfected with NEU1-HA/PPCA (1:2) and CD36 were washed three times in PBS, resuspended in 1 mL cold TEM buffer (75 mM Tris, 2 mM EDTA, 12 mM MgCl_2_, protease inhibitor cocktail, 10 mM NaF, 2 mM Na_3_VO_4_, pH 7.5) containing 1% CHAPS. After sonication, lysates were centrifuged at 600*g* for 10 min to remove nuclei and non-lysed cells. Samples were solubilized during 3 h at 4 °C under gentle end-over-end mixing. After centrifugation at 20,000*g* during 45 min at 4 °C, the supernatant was recovered. Immunoprecipitations were performed using 2 µg of rabbit monoclonal anti-HA or 1 µg mouse monoclonal anti-CD36 pre-adsorbed on protein G Sepharose beads for 2 h at 4 °C.

For macrophages, crude membranes were prepared. Cells were washed three times in PBS and resuspended in 1 mL cold TEM buffer without detergent. After sonication, lysates were centrifuged at 600*g* for 10 min to remove nuclei and non-lysed cells and crude membranes were pelleted by centrifugation at 20,000*g* during 45 min at 4 °C. The supernatant, corresponding to the cytosol fraction, was recovered. Membrane proteins were then solubilized from crude membrane pellets in TEM buffer containing 1% CHAPS (3 h, 4 °C) and centrifuged at 20,000*g* (45 min, 4 °C). The supernatant, corresponding to solubilized crude membrane proteins, was recovered. Efficient cell fractionation was confirmed by evaluating the partitioning of β_2_-integrin between cytosol and crude membrane fractions by Western blot. Immunoprecipitations were then performed on crude membrane fractions using 2 µg of rabbit polyclonal anti-NEU1 or 1 µg mouse monoclonal anti-CD36 pre-adsorbed on protein G Sepharose beads for 2 h at 4 °C. After several washes, immunoprecipitated proteins were eluted with SDS-PAGE loading buffer and subjected to SDS-PAGE and immunoblotting. Immunoblottings were performed using the indicated antibodies and immunoreactivity was revealed using HRP-conjugated secondary antibodies (1/10,000) as described above.

### Lectin blotting and lectin pulldown

Lectin blotting was performed using the DIG glycan differentiation kit (Roche) according to the manufacturer’s instructions and as described previously [5]. Briefly, after immunoprecipitation of CD36 from transfected COS-7 cells, nitrocellulose membranes were probed with either digoxigenin-labeled *Sambucus nigra* agglutinin (SNA) which binds specifically to sialic acids terminally linked to galactose or *N*-acetylgalactosamine (α-2,6), or digoxigenin-labeled *Maackia amurensis* agglutinin (MAA) which binds to galactose (α-2,3). Detection of lectin binding to the membranes was revealed by anti-digoxigenin antibodies coupled to alkaline phosphatase. As described above, detection of immunoprecipitated CD36 was revealed by a mouse monoclonal anti-CD36 (1/500) followed by HRP-conjugated anti-mouse (1/10,000).

Lectin pulldown was performed on transfected COS-7 cells or macrophages stimulated, or not, with kE (50 µg/mL), kE/V14 (1:2 molar ratio) or V14 alone for 1 h at 37 °C. Cells were washed three times in PBS and resuspended in 1 mL cold Tris/NaCl buffer (100 mM Tris, 80 mM NaCl, protease inhibitor cocktail, 10 mM NaF, 2 mM Na_3_VO_4_, pH 8) without detergent. After sonication, lysates were centrifuged at 600*g* for 10 min to remove nuclei and non-lysed cells and crude membranes were pelleted by centrifugation at 20,000*g* during 45 min at 4 °C. After solubilization in Tris/NaCl buffer containing 1% NP-40 for 3 h at 4 °C, samples were centrifuged at 20,000*g* (45 min, 4 °C) and the supernatant (solubilized crude membrane proteins) was recovered. For each condition, equal amounts of membrane proteins were incubated with 50 μg/mL biotinylated SNA (overnight, 4 °C). Streptavidin agarose beads were then added for 1 h at 4 °C. The beads were washed once with TBS/1% Triton X-100 and twice with TBS/0.5% Triton X-100, and directly resuspended in SDS-PAGE loading buffer, boiled and subjected to SDS-PAGE and immunoblotting. Immunoblottings were performed using mouse monoclonal anti-CD36 (1/500) and immunoreactivity was revealed using HRP-conjugated anti-mouse (1/10,000) as described above.

### Uptake of oxLDL

Uptake of DiI-oxLDL was studied as described previously [28]. Adherent macrophages in 24-well plates (250,000/well) were incubated, or not, with kE (50 µg/mL), DANA (400 µM), DANA/kE, kE/V14 (1:2 molar ratio) or V14 alone for 1 h at 37 °C. Then, cells were incubated with 10 µg/mL DiI-oxLDL for 4 h at 37 °C. Thereafter, cells were washed three times with PBS containing 2 mg/mL BSA, twice with PBS and fixed with 2% paraformaldehyde in PBS for 15 min. After washes, coverslips were mounted and visualized with an invert fluorescent microscope (AXIO, Zeiss). The main gray value per field was calculated using the “Integrated Density” parameter of Image J software from ten different fields per condition.

### Statistical analysis

Results are expressed as mean ± SEM. Statistical significance was evaluated using paired or unpaired Student’s *t* test, or one-way ANOVA followed by a Dunnett’s multiple comparisons test, and *p* values of less than 0.05 were considered as statistically significant.

## Results and discussion

Although NEU1 sialidase has been referred to as a lysosomal member of the sialidase family [29], accumulative data from the last 10 years have shown that NEU1 is also present at the plasma membrane of cells and regulates a myriad of membrane glycoproteins by desialylation such as the integrin beta 4 [30], TLR4 [3], Trk A [4], PDGF-BB and IGF receptors [31], EGF and MUC1 receptors [32], and CD31 [20]. This novel role for NEU1 as regulator of membrane receptor sialylation offers new dimensions in the regulation of cell signaling and modulation of biological functions [2]. Moreover, NEU1 was proposed at the center of larger signaling platforms involving G-protein-coupled receptors, a matrix metalloproteinase (MMP-9) and other receptors such as receptor tyrosine kinases or TLR4 [6, 33, 34]. At the plasma membrane, NEU1 is also part of the ERC and is involved in elastogenesis and signal transduction through this receptor [7–9], and in the biological effects mediated by EDP in atherosclerosis [13], thrombosis [35], and development of insulin resistance [5]. Consequently, NEU1 now emerges not only as a catabolic enzyme but also as a key actor involved in cell signaling regulation, membrane receptor activation and vascular diseases. Deciphering the protein complexes that associated with NEU1 at the plasma membrane of cells will help in better understanding new roles played by this sialidase in biological processes. In the current study, a proteomic approach was developed to purify and identify new interaction partners of NEU1 sialidase at the plasma membrane of macrophages, critical players of atherosclerosis.

### Settings of the proteomic approach in COS-7 cells overexpressing NEU1

**Fig. 1:**
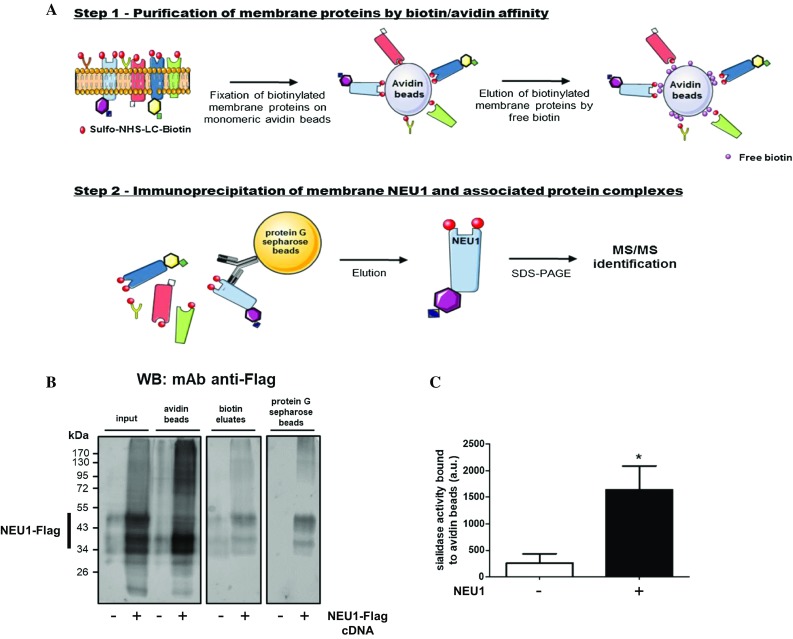
Workflow of the proteomic approach and validation of the purification procedure. **a** The approach uses a two-step purification. The first step involves biotinylation of plasma membrane proteins from adherent COS-7 cells overexpressing NEU1-Flag with the non-permeable reagent EZ-Link^®^ sulfo-NHS-LC-biotin. After cell lysis, protein lysates were incubated with monomeric avidin agarose beads to retain biotinylated plasma membrane proteins. After elution by free biotin, recovered proteins were subjected to the second step of purification which consists of immunoprecipitation of NEU1 and associated interaction partners using polyclonal rabbit anti-NEU1 antibodies. Bound material was then eluted from beads, fractionated by SDS-PAGE and stained by Coomassie blue. After systematic excision from the gel and in-gel trypsin digestion, the extracted peptides were analyzed by nano-LC–ESI MS/MS using an Orbitrap mass spectrometer. **b** COS-7 cells overexpressing human NEU1-Flag were subjected to the purification procedure (**a**) and the presence of NEU1-Flag at each step of the purification was checked by Western blot using a mouse monoclonal anti-Flag antibody (1/1000). The figure is representative of three independent experiments. NEU1-Flag−, non-transfected cells; NEU1-Flag+, cells co-transfected with NEU1-Flag and PPCA. **c** Sialidase activity associated with monomeric avidin agarose beads was measured using 200 µM of 2-O-(*p*-nitrophenyl)-α-d-*N*-acetylneuraminic acid substrate in 20 mM MES at pH 4.5. Results are expressed as mean ± SEM of four independent experiments and statistical analysis was performed by Student’s *t* test (*p < 0.05). *NEU1−* non-transfected cells; *NEU1+* cells co-transfected with NEU1 and PPCA

As NEU1 is expressed both at the plasma membrane and intracellularly, mainly in lysosomes, a two-step purification procedure was used to specifically purify plasma membrane NEU1 and its interacting partners. As illustrated in Fig. 1a, the first step involved biotinylation of plasma membrane proteins from COS-7 cells overexpressing NEU1-Flag with the non-permeable reagent EZ-Link^®^ sulfo-NHS-LC-biotin. After cell lysis, protein lysates were incubated with monomeric avidin agarose beads to retain biotinylated plasma membrane proteins. After elution by free biotin, recovered proteins were subjected to immunoprecipitation using polyclonal rabbit anti-NEU1 antibodies. Bound material was then eluted from beads, fractionated by SDS-PAGE and stained by Coomassie blue. After systematic excision from the gel and trypsin digestion, the extracted peptides were analyzed by LC–MS/MS. Settings of the approach were first performed from small-scale purifications using COS-7 cells overexpressing NEU1-Flag and PPCA, as previously reported [16], and the presence of NEU1-Flag was checked at each step of the procedure by Western blot analysis (Fig. 1b). NEU1-Flag was detected in the starting material (input), bound onto avidin beads (avidin beads), in the free biotin eluates (biotin eluates) and finally recovered at the end of the procedure after elution from protein G Sepharose beads with Laemmli buffer (protein G Sepharose beads). As shown previously, transient expression of NEU1 gives rise to multiple protein species of molecular weight between 40- and 55-kDa due to differential glycosylations of NEU1 [16, 36].

Sialidase activity bound to monomeric avidin beads was next measured using the 2-O-(*p*-nitrophenyl)-α-d-*N*-acetylneuraminic acid as substrate. To avoid any potential interference with the Flag tag, COS-7 cells overexpressing non-tagged NEU1 were subjected to the first step of the purification procedure and monomeric avidin beads were directly resuspended in 20 mM MES buffer (pH 4.5) containing 200 µM of substrate. As shown in Fig. 1c, sialidase activity of the avidin bead fractions was 6.4-fold higher for COS-7 cells overexpressing NEU1 compared to non-transfected cells. Taken together, these results demonstrated that plasma membrane NEU1 was readily recovered at the end of the two-step purification and remained functional before immunoprecipitation with anti-NEU1 antibodies.

### Validation of the proteomic approach in human macrophages endogenously expressing NEU1

The whole protocol was then applied to human macrophages whose ability to produce inflammatory cytokines and proteases, to form foam cells and their involvement in efferocytosis and autophagy make macrophages critical actors in the development of local inflammatory responses and atherosclerotic lesion growth [37]. Differentiation of monocytes into macrophages is associated with increase in NEU1 expression and its translocation from the lysosomes to the cell surface, concomitant with a 12- to 14-fold increase in sialidase activity [26, 38]. Interestingly, cell surface α2-3- and α2-6-linked sialic acids decreased after monocyte differentiation into macrophages, which is in accordance with the elevated activity of endogenous NEU1 sialidase [39]. Moreover, we and others have demonstrated that NEU1 sialidase plays a key role in monocyte/macrophage functions such as phagocytosis, cytokine release [26, 40], monocyte migration and ROS production in response to EDP [13]. Herein, macrophages were obtained by differentiation from the human monocytic THP-1 cell line, a well-known model to study monocyte/macrophage functions, mechanisms and signaling pathways, and which produce similar response patterns compared to human peripheral blood mononuclear cells [41, 42]. Differentiation of monocytes by phorbol-12-myristate-13-acetate (PMA) has been shown to produce M0 macrophages which had 20% and 30% reduction in α2-3- and α2-6-linked sialic acids at their cell surface, respectively [39]. Interestingly, polarization to M1 or M2 subtypes had no further effect on the membrane sialylation level [39], suggesting that the main modifications occurring at the levels of NEU1 and membrane sialidase activity occur during the differentiation into M0 macrophages.

**Fig. 2:**
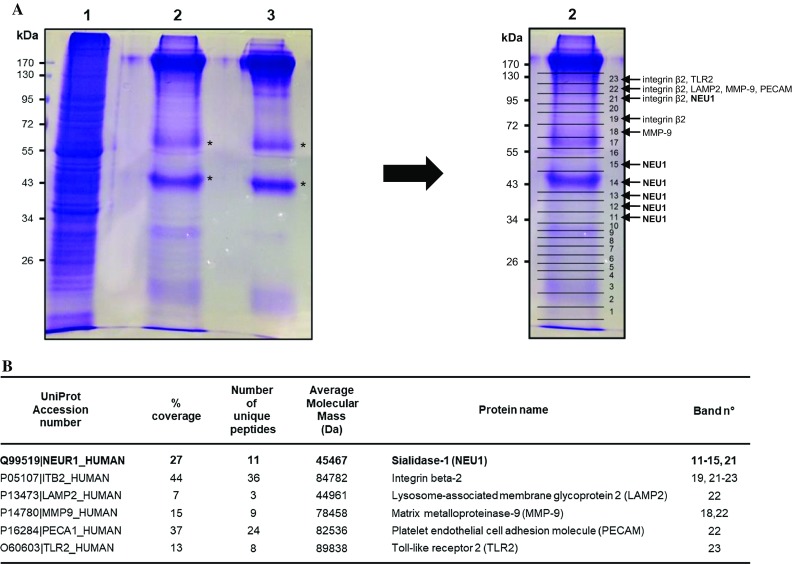
Large-scale purification of membrane NEU1 and identification of already know interaction partners. **a** Left panel: 4.8 × 10^8^ macrophages differentiated from THP-1 cells were submitted to the purification protocol and proteins recovered at the end of the purification were separated by SDS-PAGE and stained by Coomassie Blue (lane 2). Lane 1, protein lysate (starting material); lane 3, proteins recovered after elution with Laemmli buffer of protein G Sepharose beads coated with anti-NEU1 antibodies only. Asterisks, non-specific protein bands recovered in both lanes 2 and 3. Right panel: as illustrated for lane 2, lanes were excised into 23 bands and in-gel digested by trypsin for analysis by mass spectrometry. **b** Identification by LC–MS/MS of already know interaction partners of membrane NEU1. Trypsin-digested protein bands were analyzed by nano-LC–ESI MS/M, and proteins identified with Mascot software in Swiss-Prot and trEMBL databases

The ultimate goal of such a two-step purification procedure is the purification of sufficient amounts of the target protein to identify associated proteins by mass spectrometry analysis. To reach this goal, about 480 million macrophages were used and submitted to the whole procedure. Proteins recovered at the end of the purification were separated by one-dimensional gel electrophoresis and stained by Coomassie Blue (Fig. 2a, left panel, lane 2). As a control, protein G Sepharose beads coated with anti-NEU1 antibodies were also subjected to one-dimensional gel electrophoresis and Coomassie Blue staining to visualize bands corresponding to anti-NEU1 antibodies and to identify the non-specific proteins provided by the coated beads alone (Fig. 2a, left panel, lane 3). Two major bands migrating around 50- and 60-kDa, respectively, were recovered in both lanes (Fig. 2a, left panel, asterisks). In contrast to the coated beads alone (lane 3), several additional bands were also detected over the whole lane 2 after the purification procedure. As illustrated for lane 2 (Fig. 2a, right panel), lanes were systematically excised into 23 bands, in-gel digested with trypsin, and the resulting peptides were analyzed by mass spectrometry and identified from UniProt/Swiss-Prot databases. As expected, MS/MS identifies the two major bands migrating around 50- and 60-kDa (Fig. 2a, left panel, asterisks) as IgG and serum albumin. Importantly, MS/MS identification confirmed the presence of NEU1 in lane 2 (Fig. 2b) between 34- and 55-kDa and around 95-kDa in agreement with its glycosylation profile and its ability to form dimeric structures [16, 36]. In addition, already known interacting partners of NEU1 and/or proteins already known to be regulated by NEU1 were unambiguously identified such as integrin beta-2, lysosome-associated membrane glycoprotein 2 (LAMP2), matrix metalloproteinase-9 (MMP-9), platelet endothelial cell adhesion molecule (PECAM) and toll-like receptor 2 (TLR2) (Fig. 2b). Taken together, these data validate the procedure used.

### Identification of potential interaction partners of membrane NEU1 in human macrophages

In addition to the identification of already known interacting partners of NEU1 (Fig. 2b), 71 unique proteins were unambiguously identified by mass spectrometry from two independent experiments (Table 1). These candidates that remain to be confirmed in further experiments, showed localization to different subcellular compartments (plasma membrane, cytosol and cytoskeleton) and could be divided into five distinct groups: membrane-bound, transmembrane, signaling, and cytoskeleton proteins, and proteins involved in vesicle trafficking. This indicates that NEU1 may associate with different protein complexes at the plasma membrane of macrophages, as originally proposed by the group of Szewczuk and co-workers by the discovery of membrane signaling platforms involving NEU1, MMP-9, a G-protein-coupled receptor (GPCR) and either the insulin receptor or TLR4 [6, 33, 34, 43]. Formation of such complexes has been reported to play important role in reciprocal receptor transactivation [6, 33, 34, 43]. As exemplified for GPCR-associated protein complexes [44], protein complexes are dynamic assemblies and their composition may change according to the presence of agonists. It is tempting to speculate that such modulation may occur within NEU1-associated complexes under different pathophysiological contexts such as during inflammatory conditions.

**Table 1:** Protein identification by LC–MS/MS

UnitProt accession number	Protein name	% coverage	Number of unique peptide	Average molecular mass (kDa)	Band no.
Membrane-bound proteins					
Q96CX2|KCD12_HUMAN	BTB/POZ domain-containing protein KCTD12	16	4	35.7	7
Q9UGN4|CLM8_HUMAN	CMRF35-like molecule 8	9	2	33.2	16, 17
O75131|CPNE3_HUMAN	Copine-3	6	2	60.1	13
Q99961|SH3G1_HUMAN	Endophilin-A2	12	4	41.5	11
P54753|EPHB3_HUMAN	Ephrin type-B receptor 3	2	2	110.3	5
Q3B8N2|LEG9B_HUMAN	Galectin-9B	10	3	39.7	1, 21, 23
Q6DKI2|LEG9C_HUMAN	Galectin-9C	10	3	39.6	1, 21, 23
P48307|TFPI2_HUMAN	Tissue factor pathway inhibitor 2	10	2	26.9	9, 10
Transmembrane proteins					
P20273|CD22_HUMAN	B-cell receptor CD22	11	8	95.3	22
P21730|C5AR1_HUMAN	C5a anaphylatoxin chemotactic receptor 1	8	2	39.3	12, 22
P08962|CD63_HUMAN	CD63 antigen	7	2	25.6	15
P60033|CD81_HUMAN	CD81 antigen	12	2	25.8	2
P16671|CD36_HUMAN	Platelet glycoprotein 4 (CD36**)**	14	5	53.1	20, 21
Q15438|CYH1_HUMAN	Cytohesin-1	19	2	46.4	14
Q8WXG9|GPR98_HUMAN	G-protein-coupled receptor 98	1	3	693.1	19
P18462|1A25_HUMAN	HLA class I histocompatibility antigen, A-25 alpha chain	8	2	41.2	15
P30450|1A26_HUMAN	HLA class I histocompatibility antigen, A-26 alpha chain	8	2	41.1	15
P30512|1A29_HUMAN	HLA class I histocompatibility antigen, A-29 alpha chain	8	2	40.9	15
P16189|1A31_HUMA	HLA class I histocompatibility antigen, A-31 alpha chain	8	2	41.0	15
P10314|1A32_HUMAN	HLA class I histocompatibility antigen, A-32 alpha chain	17	4	41.0	15, 16
P16190|1A33_HUMAN	HLA class I histocompatibility antigen, A-33 alpha chain	8	2	40.9	15
P30453|1A34_HUMAN	HLA class I histocompatibility antigen, A-34 alpha chain	8	2	41.1	15
P30456|1A43_HUMAN	HLA class I histocompatibility antigen, A-43 alpha chain	8	2	41.0	15
P30457|1A66_HUMAN	HLA class I histocompatibility antigen, A-66 alpha chain	8	2	41.1	15
P01891|1A68_HUMAN	HLA class I histocompatibility antigen, A-68 alpha chain	8	2	40.9	15
P30459|1A74_HUMAN	HLA class I histocompatibility antigen, A-74 alpha chain	17	4	40.9	15, 16
P04222|1C03_HUMAN	HLA class I histocompatibility antigen, Cw-3 alpha chain	27	2	40.9	13
P30505|1C08_HUMAN	HLA class I histocompatibility antigen, Cw-8 alpha chain	44	2	40.8	13
P23467|PTPRB_HUMAN	Receptor-type tyrosine-protein phosphatase beta	1	2	224.3	12
Q12913|PTPRJ_HUMAN	Receptor-type tyrosine-protein phosphatase eta	3	3	145.9	20
Signaling					
P31947|1433S_HUMAN	14–3–3 protein sigma	5	2	27.8	13
Q99996|AKAP9_HUMAN	A-kinase anchor protein 9	0	2	453.7	13
Q12802|AKP13_HUMAN	A-kinase anchor protein 13	0	3	307.6	3, 4, 7, 10, 13, 15
P62158|CALM_HUMAN	Calmodulin	41	8	16.8	1
P01112|RASH_HUMAN	GTPase HRas	12	2	21.3	10
P01111|RASN_HUMAN	GTPase NRas	12	2	21.2	10
Q9UMX6|GUC1B_HUMAN	Guanylyl cyclase-activating protein 2	32	7	23.4	1, 3, 5, 9
Q16539|MK14_HUMAN	Mitogen-activated protein kinase 14	19	5	41.3	13
Q9Y314|NOSIP_HUMAN	Nitric oxide synthase-interacting protein	30	7	33.2	11
P49593|PPM1F_HUMAN	Protein phosphatase 1F	44	20	49.8	16, 17
Q13188|STK3_HUMAN	Serine/threonine-protein kinase 3	10	4	56.3	18
O00506|STK25_HUMAN	Serine/threonine-protein kinase 25	12	2	48.1	17
Q9P289|STK26_HUMAN	Serine/threonine-protein kinase 26	19	4	46.5	15
Q86Y07|VRK2_HUMAN	Serine/threonine-protein kinase VRK2	7	3	58.1	16
Q9UKE5|TNIK_HUMAN	TRAF2 and NCK-interacting protein kinase	8	3	154.9	17, 19
P07948|LYN_HUMAN	Tyrosine-protein kinase Lyn	5	2	58.6	16
Q12866|MERTK_HUMAN	Tyrosine-protein kinase Mer	8	7	110.2	23
Q9UGJ0|AAKG2_HUMAN	5′-AMP-activated protein kinase subunit gamma-2	3	2	63.1	13
Q96JB6|LOXL4_HUMAN	Lysyl oxidase homolog 4	7	4	84.5	19
P01033|TIMP1_HUMAN	Metalloproteinase inhibitor 1	11	2	23.2	5
Cytoskeleton					
O15143|ARC1B_HUMAN	Actin-related protein 2/3 complex subunit 1B	20	6	41.0	12
P59998|ARPC4_HUMAN	Actin-related protein 2/3 complex subunit 4	21	4	19.7	1
Q562R1|ACTBL_HUMAN	Beta-actin-like protein 2	39	2	42.0	12, 13
Q86UX7|URP2_HUMAN	Fermitin family homolog 3	6	2	76.0	18, 19, 22
Q9UGP4|LIMD1_HUMAN	LIM domain-containing protein 1	11	5	72.2	19
P19105|ML12A_HUMAN	Myosin regulatory light chain 12A	30	5	19.8	112
O14950|ML12B_HUMAN	Myosin regulatory light chain 12B	30	5	19.8	1
P24844|MYL9_HUMAN	Myosin regulatory light polypeptide 9	17	3	19.8	1
P07737|PROF1_HUMAN	Profilin-1	19	2	15.1	13
Q13464|ROCK1_HUMAN	Rho-associated protein kinase 1	3	4	158.2	23
Q71U36|TBA1A_HUMAN	Tubulin alpha-1A chain	9	3	50.1	1, 2, 10, 11
Q6PEY2|TBA3E_HUMAN	Tubulin alpha-3E chain	28	3	49.9	13
Q9BVA1|TBB2B_HUMAN	Tubulin beta-2B chain	5	2	49.9	11
P04350|TBB4A_HUMAN	Tubulin beta-4A chain	32	4	49.6	11, 12
Vesicle trafficking					
Q92928|RAB1C_HUMAN	Putative Ras-related protein Rab-1C	37	5	22.1	1, 4
Q92546|RGP1_HUMAN	RAB6A-GEF complex partner protein 2	12	4	42.5	13, 14
Q5HYI8|RABL3_HUMAN	Rab-like protein 3	22	6	26.4	7
Q9H0U4|RAB1B_HUMAN	Ras-related protein Rab-1B	37	5	22.2	1, 4
Q14964|RB39A_HUMAN	Ras-related protein Rab-39A	26	5	25.0	1
O43752|STX6_HUMAN	Syntaxin-6	9	2	29.2	10

Trypsin-digested protein bands from two independent experiments were analyzed by nano-LC-ESI MS/MS and proteins identified with Mascot software in Swiss-Prot and trEMBL databases

Among the proteins identified in the two independent experiments, the class B scavenger receptor CD36, also known as platelet glycoprotein 4, was identified with 5 unique peptides covering 14% of the whole amino acid sequence of CD36 (Table 1). This plasma membrane glycoprotein heavily modified post-translationally by N-linked glycosylation with 9 out the 10 putative glycosylation sites being modified [17]. CD36 is expressed in monocytes, macrophages, and also in platelets, epithelial, endothelial and smooth muscle cells, binds many different ligands and is involved in several diseases scenarios such as inflammation, atherosclerosis, thrombosis and angiogenesis [45, 46]. In atherosclerosis, CD36 plays significant roles including foam cell formation, release of inflammatory mediators, macrophage trapping and thrombosis [46].

### Validation of the interaction between NEU1 and CD36 and functional consequences on the sialylation level of CD36

**Fig. 3:**
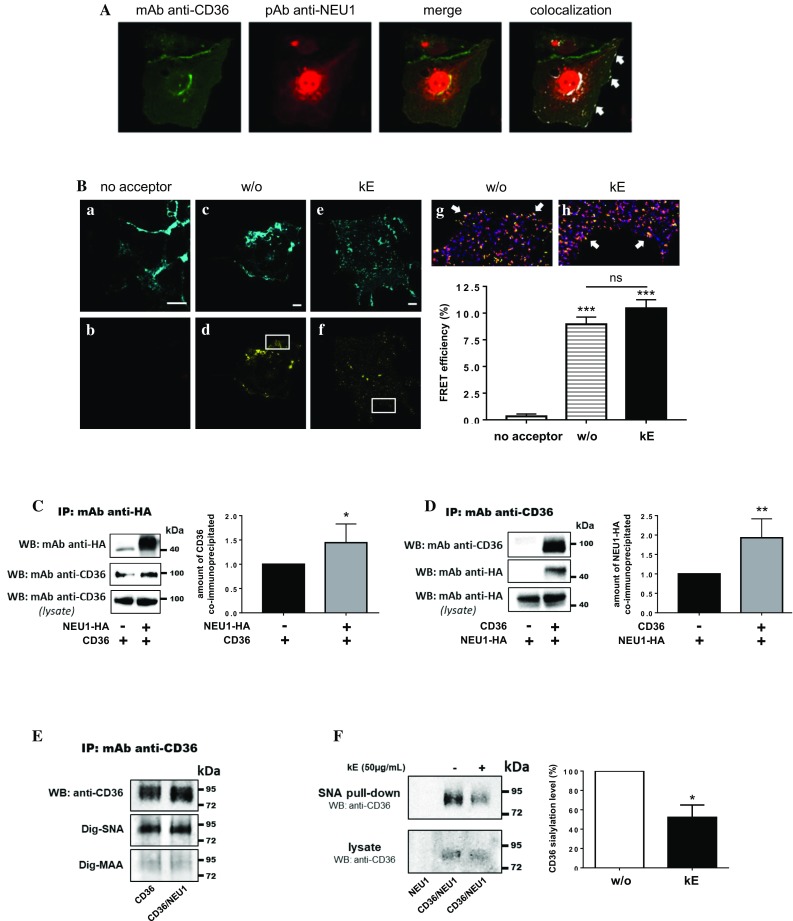
Validation of the interaction between NEU1 and CD36 in COS-7 cells and functional consequences on the sialylation level of CD36. **A** Colocalization of NEU1 and CD36 at the cell surface of COS-7 cells co-expressing human NEU1 and human CD36 by confocal microscopy acquisitions. Areas of colocalization at the plasma membrane are indicated by white arrows. Images are representative of two independent experiments. **B** FRET experiments in COS-7 cells expressing CD36-Teal (energy donor) or CD36-Teal and NEU1-YFP (energy acceptor). Confocal images after spectral unmixing for the donor (**a**, **c**, **e**) and the acceptor (**b**, **d**, **f**) in a cell incubated (**e**, **f**), or not (**a**–**d**), with kE (50 µg/mL, 1 h), or without acceptor (**a**, **b**). Zoom area of calculated FRET efficiency are presented in Fire LUT for each condition (**g**, **h**). White arrows indicate areas of higher FRET efficiency. The graph represents the mean FRET efficiency ± SEM of three independent experiments (three cells/experiment). For each cell, thirty regions of interest of 0.62 µm^2^ contouring the cell were chosen. Statistical analysis was performed by Student’s *t* test (***p < 0.001 versus no acceptor; *ns* non-significant). Scale bar: 10 µm. **C** Left panel: NEU1-HA was immunoprecipitated with a rabbit monoclonal anti-HA antibody from whole lysate of COS-7 cells expressing CD36 (NEU1-HA−/CD36+) or co-expressing human NEU1-HA and CD36 (NEU1-HA+/CD36+). Co-immunoprecipitation of CD36 was monitored by Western blot using a mouse monoclonal anti-CD36 antibody (1/500). The figure is representative of six independent experiments. Right panel: blot quantification by densitometry analysis. Results are expressed as mean ± SEM of six independent experiments and normalized to NEU1-HA−/CD36+ condition. Statistical analysis was performed by Student’s *t* test (*p < 0.05). **D** Left panel: CD36 was immunoprecipitated with a mouse monoclonal anti-CD36 antibody from whole lysate of COS-7 cells expressing NEU1-HA (CD36−/NEU1-HA+) or co-expressing NEU1-HA and CD36 (CD36+/NEU1-HA+). Co-immunoprecipitation of NEU1-HA was monitored by Western blot using a rabbit monoclonal anti-HA antibody (1/1000). The figure is representative of four independent experiments. Right panel: blot quantification by densitometry analysis. Results are expressed as mean ± SEM of four independent experiments and normalized to CD36−/NEU1-HA+ condition. Statistical analysis was performed by Student’s *t* test (**p < 0.01). **E** Lectin blotting after immunoprecipitation of CD36 from whole lysates of COS-7 cells expressing CD36 (CD36) or co-expressing CD36 and NEU1 (CD36/NEU1). Nitrocellulose membranes were incubated with digoxigenin-labeled SNA or MAA lectins followed by anti-digoxigenin antibodies coupled to alkaline-phosphatase according to the manufacturer’s instructions. Detection of immunoprecipitated CD36 was revealed as described above. The figure is representative of two independent experiments. **F** Left panel: SNA pulldown of crude membrane preparations of COS-7 cells expressing NEU1 (NEU1) or co-expressing CD36 and NEU1 (CD36/NEU1), and incubated, or not, with kE (50 µg/mL, 1 h). For each condition, equal amount of proteins was used. The amount of sialylated CD36 recovered after SNA pulldown was evaluated by Western blot using a mouse monoclonal anti-CD36 antibody as described above. The figure is representative of three independent experiments. Right panel: quantification of the sialylation level of CD36 (pulldown/lysate ratio) by densitometry analysis. The sialylation level of CD36 was normalized to the control condition (without kE, w/o). Results are expressed as mean ± SEM of three independent experiments and statistical analysis was performed by Student’s *t* test (*p < 0.05)

Validation of the interaction between NEU1 and CD36 was first performed in COS-7 cells overexpressing non-tagged NEU1 and CD36. As shown in Fig. 3A, transfected NEU1 localized at the plasma membrane and inside the cells, mainly in lysosomes, as previously described [16]. Transfected CD36 was also detected both intracellularly and at the plasma membrane and showed colocalization with NEU1. Confocal FRET imaging analysis in COS-7 cells transfected with CD36-Teal (energy donor) and NEU1-YFP (energy acceptor) provided additional evidence that both proteins were in sufficient proximity to interact with each other at the plasma membrane (Fig. 3B). In cells transfected with CD36-Teal alone, excitation of the donor at 458 nm led to a residual fluorescence emission at 527 nm, corresponding to the wavelength of acceptor emission. This cross-talk was corrected using FRET analysis based on spectral unmixing (see “Materials and methods”), and non-specific FRET efficiency was estimated at 0.3 ± 0.2%. When cells were co-transfected with both CD36-Teal and NEU1-YFP, FRET efficiency in the colocalization areas at the plasma membrane was significantly increased to 9.0 ± 0.7%. At the plasma membrane, NEU1 is part of the ERC and is required for elastogenesis and signal transduction through this receptor [7–9]. NEU1 constitutes the catalytic subunit of the receptor [7, 9] and stimulation of cells by EDP that originate from elastin degradation during vascular aging [12], triggers NEU1 sialidase activity and signal transduction. We therefore evaluated the effects of EDP, derived from organo-alkaline hydrolysate of insoluble bovine purified elastin (kappa-elastin, kE), on FRET efficiency between CD36-Teal and NEU1-YFP. When cells were stimulated by kE (50 µg/mL), FRET efficiency in the colocalization areas at the plasma membrane was 10.5 ± 0.8% (Fig. 3B). Although not significant, this slight increase in FRET efficiency observed between non-stimulated (w/o) and stimulated (kE) cells, may suggest conformational changes within the CD36-NEU1 complex that lead to reorientation of the FRET pair and optimization of energy transfer. Interaction between NEU1 and CD36 was further confirmed by co-immunoprecipitation from whole cell lysates. Immunoprecipitation of NEU1-HA in COS-7 cells co-expressing NEU1-HA and CD36 led to a significant increase by 44.3 ± 15.8% of the amount of co-immunoprecipitated CD36 compared to cells expressing CD36 alone (Fig. 3C). Reciprocally, immunoprecipitation of CD36 in cells co-expressing CD36 and NEU1-HA led to a significant increase by 93.0 ± 24.4% of the amount of co-immunoprecipitated NEU1-HA compared to cells expressing NEU1-HA alone (Fig. 3D).

Although much is known about the glycosylation status of CD36 [17], less is known about its sialylation. We therefore assessed the presence of sialic acids terminally linked to galactose or N-acetylgalactosamine (α2-6) or to galactose (α2-3) on immunoprecipitated CD36 from transfected COS-7 cells using the digoxigenin-coupled *Sambucus nigra* (Dig-SNA) or *Maackia amurensis* (Dig-MAA) lectins, respectively. As shown in Fig. 3E, and in contrast to Dig-MAA, a strong labeling was observed with the Dig-SNA lectin at molecular weight corresponding to immunoprecipitated CD36 from cells overexpressing CD36 or CD36 and NEU1. These results suggested that CD36 may be modified predominantly with α-2,6- rather than α-2,3-sialylation. We next used a lectin pulldown assay with biotinylated SNA to evaluate the effects of kE on the α2-6 sialylation level of CD36. As depicted in Fig. 3F, the amount of sialylated CD36 recovered after SNA pulldown was significantly decreased by 47.8 ± 12.7% after incubation of COS-7 cells with kE (50 µg/mL). Taken together, these data demonstrated that regulation of NEU1 sialidase activity by EDP had functional consequences on the sialylation level of CD36.

**Fig. 4:**
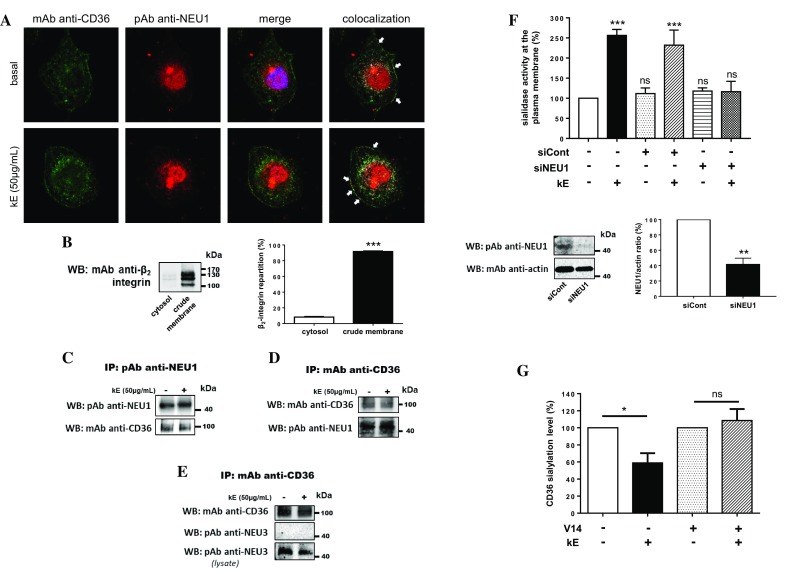
Validation of the interaction between NEU1 and CD36 in human macrophages and functional consequences on the sialylation level of CD36. **a** Colocalization of NEU1 and CD36 at the cell surface of macrophages differentiated from THP-1 cells, and stimulated, or not, with kE (50 µg/mL, 1 h), by confocal microscopy acquisitions. Areas of colocalization at the plasma membrane are indicated by white arrows. Images are representative of two independent experiments. **b** Left panel: distribution of the β_2_-integrin between the cytosol and crude membrane fractions (30 µg each) of macrophages by Western blot using a mouse monoclonal anti β_2_-integrin (1/500). The figure is representative of three independent experiments. Right panel: blots quantification by densitometry analysis. Results are expressed as mean ± SEM of three independent experiments and statistical analysis was performed by Student’s *t* test (***p < 0.001). **c** NEU1 was immunoprecipitated with a rabbit polyclonal anti-NEU1 antibody from crude membrane preparations of macrophages and co-immunoprecipitation of CD36 was monitored by Western blot using a mouse monoclonal anti-CD36 antibody. The figure is representative of three independent experiments. **d** CD36 was immunoprecipitated with a mouse monoclonal anti-CD36 antibody from crude membrane preparations of macrophages and co-immunoprecipitation of NEU1 was monitored by Western blot using a rabbit polyclonal anti-NEU1 antibody (1/500). The figure is representative of two independent experiments. **e** CD36 was immunoprecipitated with a mouse monoclonal anti-CD36 antibody from whole lysate of macrophages and co-immunoprecipitation of NEU3 was monitored by Western blot using a rabbit polyclonal anti-NEU3 antibody (1/500). The figure is representative of three independent experiments. **f** Up panel: sialidase activity at the plasma membrane of adherent macrophages was measured using 400 µM of 2′-(4-methylumbelliferyl)-α-d-*N*-acetylneuraminic acid substrate in 20 mM CH_3_COONa (pH 6.5) before and after incubation with kE (50 µg/mL, 2 h). Macrophages were either non-transfected or transfected with 50 nM negative control siRNA (siCont) or NEU1 siRNA (siNEU1). Results are expressed as mean ± SEM of three to nine independent experiments and normalized to the control (non-transfected, without kE). Statistical analysis was performed by one-way ANOVA followed by a Dunnett’s multiple comparisons test (***p < 0.001; *ns* non-significant). Down panel: expression level of NEU1 in macrophages transfected with 50 nM negative control siRNA (siCont) or NEU1 siRNA (siNEU1) and monitored by Western blot using a rabbit polyclonal anti-NEU1 antibody. The blot is representative of three independent experiments (left). Blots were quantified by densitometry analysis, and results expressed as mean ± SEM of three independent experiments and normalized to negative control siRNA (siCont). Statistical analysis was performed by Student’s *t* test (**p < 0.01. **g** SNA pulldown of crude membrane preparations of macrophages incubated, or not, with kE (50 µg/mL), V14 + kE (molar ratio 2:1) or V14 peptide alone for 1 h at 37 °C. For each condition, equal amount of proteins was used. The amount of sialylated CD36 recovered after SNA pulldown was evaluated and quantified as depicted in Fig. 3F. The sialylation level of CD36 was normalized to the respective control (w/o or V14) and results expressed as mean ± SEM of three to five independent experiments. Statistical analysis was performed by Student’s *t* test (*p < 0.05; *ns* non-significant)

Similar experiments were performed in human macrophages endogenously expressing both NEU1, CD36 and the ERC. As shown in Fig. 4a, colocalization of NEU1 and CD36 was clearly observed at the plasma membrane of macrophages and prior incubation with kE was without effect. Reciprocal co-immunoprecipitations were performed from crude membrane preparations of macrophages and efficient cell fractionation was first studied by evaluating β_2_-integrin distribution between the cytosol and crude membrane fractions. Densitometry analysis revealed that 91.7 ± 0.9% of β_2_-integrin was recovered in crude membrane fractions (Fig. 4b). Reciprocal co-immunoprecipitations showed that endogenous CD36 and NEU1 interacted together in macrophages (Fig. 4c, d) and that prior incubation with kE had no effect, rather suggesting a constitutive interaction between CD36 and NEU1. Moreover, we evaluated if NEU3, another membrane sialidase, could interact with CD36. Under the same experimental conditions, NEU3 failed to co-immunoprecipitate with CD36 (Fig. 4e). These results therefore strengthened the specificity of the interaction between NEU1 and CD36.

We next assessed the effect of kE on sialidase activity at the plasma membrane of macrophages as previously described for dermal fibroblasts [7]. As shown in Fig. 4f, incubation with kE significantly increased by 156.4 ± 14.7% sialidase activity at the plasma membrane. Compared to negative control siRNA, silencing NEU1 by 50 nM NEU1 siRNA totally blocked these effects of kE and definitely demonstrated involvement of NEU1 in kE-induced sialidase activity at the plasma membrane of macrophages. This NEU1-mediated increase in sialidase activity, in the presence of kE, was associated with a significant decrease by 41.1 ± 11.4% of the sialylation level of CD36 (Fig. 4g). To further confirm that this modulating effect on CD36 sialylation level involved the ERC, kE was pre-incubated with the V14 peptide (VVGSPSAQDEASPL), a synthetic peptide blocking the interaction between the GxxPG motifs contained in kE and the ERC [47, 48], prior to its incubation with cells. In these conditions, the effects of kE were blocked (Fig. 4g). Taken together, these results demonstrated that both the ERC and its NEU1 subunit were involved in these effects.

### Elastin-derived peptides potentiate oxLDL uptake in human macrophages

**Fig. 5:**
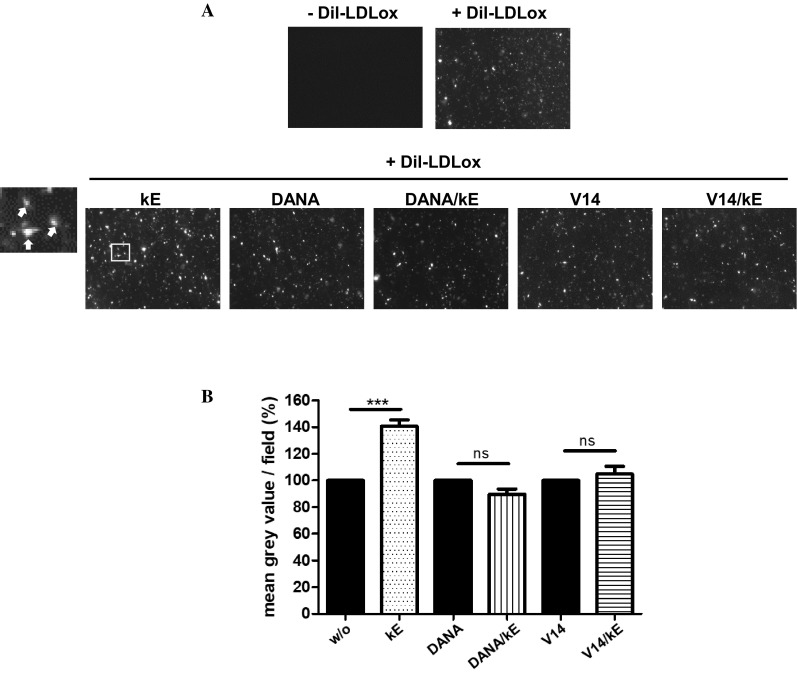
EDP increase the uptake of Dil-oxLDL in human macrophages through the ERC and its NEU1 subunit. **a** Human adherent macrophages were incubated with kE (50 µg/mL), DANA (400 µM), DANA + kE, V14 + kE (molar ratio 2:1) or V14 peptide alone for 1 h at 37 °C prior to the addition of 10 µM Dil-oxLDL (4 h, 37 °C). Cells were then washed, fixed and visualized by a fluorescent microscope. A representative field is shown. The inset corresponds to a zoom area of the kE condition and white arrows show the presence of DiI-oxLDL particles inside the cells. **b** The mean gray value per field for each condition was calculated and normalized to the respective control (w/o, DANA, V14). Results are expressed as mean ± SEM of six independent experiments (ten different fields/experiment). Statistical analysis was performed by Student’s *t* test (***p < 0.001; *ns* non-significant)

Macrophage CD36 plays a critical role in atherosclerosis through its interaction with oxidized low-density lipoprotein (oxLDL), which triggers signaling cascades for inflammatory responses, oxLDL uptake and formation of foam cell formation, which is the initial critical stage of atherosclerosis. CD36 is thought to be responsible for at least 50% of oxLDL uptake by murine and human macrophages [49–51]. To determine if EDP would affect the uptake of oxLDL in these cells, we evaluated the effects of kE on oxLDL uptake using 1,10-dioctadecyl-3,3,3030-tetramethylindocyanide percholorate (DiI)-labeled oxLDL (DiI-oxLDL) and fluorescent microscopy as described previously [28]. As shown in Fig. 5, pre-incubation of cultured macrophages with kE prior to the addition of Dil-oxLDL (10 µg/mL, 4 h) led to a significant increase in oxLDL uptake by 40.8 ± 4.7% compared to macrophages without kE (w/o). At higher magnification (Fig. 5, inset), DiI-oxLDL particles were found inside in the cells and not bound on the plasma membrane. Importantly, the V14 peptide and 2-deoxy-2,3-didehydro-*N*-acetylneuraminic acid (DANA), a sialidase inhibitor, blocked these potentiating effects of kE and demonstrated that both the ERC and NEU1 were involved in these effects. If these potentiating effects of EDP arise from their impact on the sialylation level of CD36 remains to be investigated.

In conclusion, we have identified herein a new interaction partner of plasma membrane NEU1 in macrophages, the scavenger receptor CD36. We showed that EDP, through the ERC and its catalytic subunit NEU1, regulate the sialylation level of CD36 and modulate the uptake of oxLDL in these cells. This study strengthens the key role played by this plasma membrane sialidase in macrophages and may have potential implications in the proatherogenic effects of EDP described previously in atherosclerosis [13]. It is tempting to speculate that by its ability to form dimeric structures at the plasma membrane [16], NEU1 may favor proximity and close interaction between the ERC and adjacent receptors for potential cross-talk and transactivation of receptors. By their ability to modulate the sialylation level of adjacent membrane receptors through NEU1, new roles for EDP and the ERC are anticipated in the fine-tuning of membrane receptor activation and signaling.
